# Quantitative analysis of free polyethylene glycols in PEGylated peptides using reverse-phase high-performance liquid chromatography and charged aerosol detection

**DOI:** 10.1371/journal.pone.0357292

**Published:** 2026-09-03

**Authors:** Yue Sun, Xinyue Hu, Yue Huang, Yuqin Yang, Xiaoming Zhang, Yi Li, Ping Lyu, Jing Li

**Affiliations:** 1 National Institutes for Food and Drug Control, State Key Laboratory of Drug Regulatory Science, NMPA Key Laboratory for Quality Research and Evaluation of Chemical Drugs, Beijing, China; 2 Beijing Institute of Petrochemical Technology, Beijing, China; 3 Taizhou Institute for Food and Drug Control, Taizhou, Zhejiang, China; Hamadan University of Medical Sciences, IRAN, ISLAMIC REPUBLIC OF

## Abstract

PEGylation is a well-established strategy used to enhance the pharmacokinetic and pharmacodynamic properties of peptide drugs. However, free polyethylene glycol (PEG) remains a critical process-related impurity. Conventional detection methods for nonchromophoric compounds such as PEG, which include the use of evaporative light scattering (ELS) and refractive index (RI) detectors, are limited by their low sensitivity and poor compatibility with the gradient elution. Herein, systematically optimized reverse-phase high-performance liquid chromatography (RP-HPLC) coupled with charged aerosol detection (CAD) was developed and validated for the quantification of free PEG in three representative PEGylated peptide therapeutics: PEG-loxenatide, pegmolesatide, and visepegenatide. The proposed method implements a co-optimization strategy for CAD parameters, with the power function value (PFV) applied during CAD signal acquisition and the power law(PL) setting adjusted during data processing.This dual optimization effectively linearizes the inherently nonlinear CAD response across a broad concentration range, enabling a linear relationship between PEG concentration and detector response. Method validation demonstrated satisfactory specificity, precision, linearity, accuracy, robustness, and solution stability in accordance with the applicable guidelines and pharmacopoeial requirements. Practical applicability was confirmed by analyzing multiple drug batches and accelerated stability samples.A Wilcoxon signed-rank test showed no statistically significant difference between the results obtained under the default and co-optimized settings (P > 0.05), indicating that the two settings produced comparable results under the conditions evaluated. The proposed HPLC-CAD method provides a practical analytical approach in the analysis of PEGylated peptide pharmaceuticals by achieving a linearized CAD response through PFV and PL co-optimization, providing an effective approach for free PEG quantification in routine quality control.

## 1 Introduction

Polyethylene glycol (PEG) is a class of synthetic polymer composed of repeating oxyethylene subunits, characterized by specific molecular weight distributions and hydrophilic properties. PEGs exist in linear, branched, and various other molecular configurations. Their nontoxic, nonimmunogenic, and nonantigenic nature, combined with their excellent aqueous solubility, has led to their use in pharmaceutical applications [1–3]. PEGylated peptide drugs are therapeutic agents modified via covalent conjugation between active functional groups at the PEG terminus and either amino acid side-chains or amino/carboxyl groups located at the N-/C-terminus of peptides [4,5]. PEGylation is a well-established strategy that improves therapeutic outcomes by extending drug half-life through reduced renal clearance and proteolytic degradation, while also reducing immunogenicity, increasing solubility and stability, and optimizing biodistribution for superior efficacy and less frequent dosing [6–8].

Free polyethylene glycol (free PEG) refers to PEG that is not conjugated to peptides during synthesis or degraded from the conjugate during storage. The EMA Guideline on the Development and Manufacture of Synthetic Peptides specifies that the quantification of unconjugated conjugate moieties (e.g., free PEG or linker residues) is an important quality attribute for conjugated peptide products [9]. The general monograph for pegylated recombinant protein and polypeptide products for human use [10] specifies residual PEG as a critical impurity testing parameter. This control strategy ensures batch-to-batch consistency and mitigates potential risks to patient safety. As PEG does not exhibit significant UV absorbance, universal detectors can be employed for the quantitative analysis of free PEG. Evaporative light-scattering (ELS) and refractive index (RI) detectors serve as universal detection options for compounds lacking UV chromophores [11–14]. However, ELS detectors are hindered by their nonlinear response, relatively narrow dynamic range, and significant inter-analyte response variability, with attempts to enhance their sensitivity leading to undesirable chromatographic artifacts such as spike peaks. RI detectors have low sensitivity and poor compatibility with the gradient elution due to the continuous baseline drift caused by the changing mobile phase composition [15–17]. Current strategies for the quality control of free PEG predominantly rely on UV detectors, which utilize the weak UV absorption of PEG activation groups, or ELS detectors [1,2]. Nevertheless, these conventional methods have insufficient sensitivity, rendering them incapable of detecting low levels of free PEG impurities and thereby posing significant risks to product quality and safety. Charged aerosol detection (CAD) offers distinct advantages over traditional detectors, including higher sensitivity and a more uniform response, making it highly suitable for monitoring free PEG in PEGylated pharmaceuticals [18,19].

**Fig 1:**
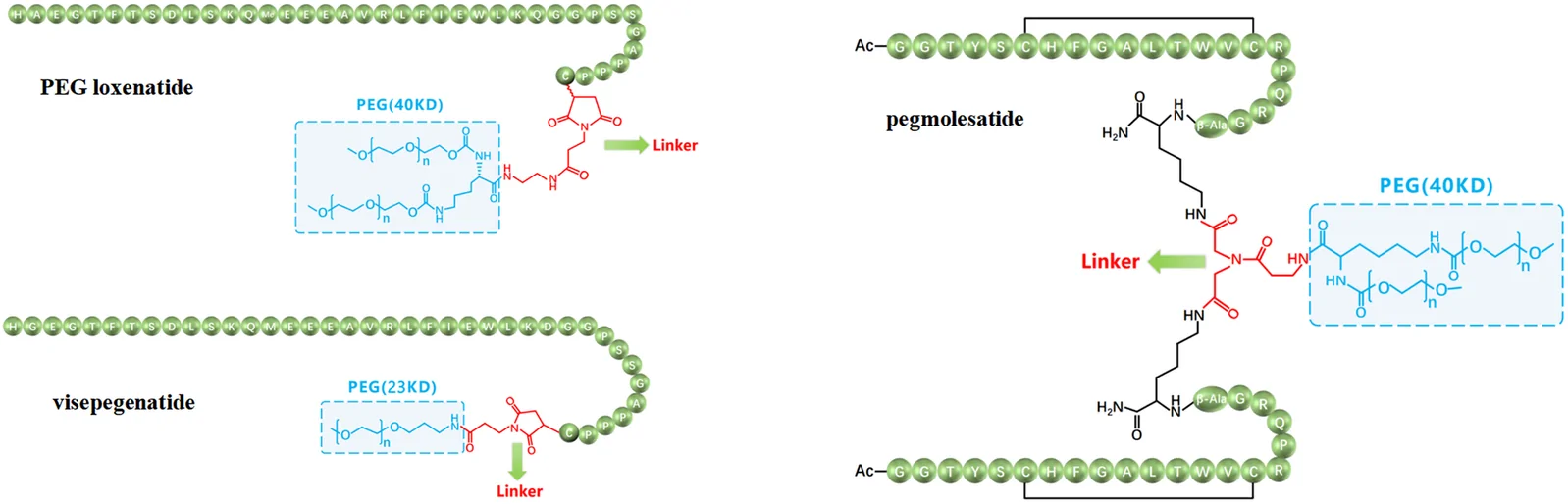
Chemical structures of PEG-loxenatide, pegmolesatide, and visepegenatide.

In this study, a reverse-phase high-performance liquid chromatography method coupled with charged aerosol detection (HPLC-CAD) was established for the determination of free PEG, which utilized three PEGylated peptide drugs, PEG-loxenatide (PEG-loxe), pegmolesatide, and visepegenatide, as model compounds (their chemical structures are shown in Fig 1). PEG-loxe is a novel hypoglycemic drug composed of macromolecules that is prepared by modifying amino acids and PEG based on the chemical structure of exenatide. Notably, it is the first long-acting GLP-1RA to be marketed in China [20,21]. Structurally, PEG-loxe is synthesized by linking two 20-kDa methoxy PEG chains via a lysine linker to generate a 40-kDa branched PEG moiety, which is subsequently functionalized with a maleimide group. This activated PEG derivative conjugates with the thiol group of the C-terminal cysteine residue of loxenatide to form a stable thioether linkage. Pegmolesatide is a synthetic, PEGylated, peptide-based erythropoiesis-stimulating agent [22,23] that is synthesized by forming an amide bond between the primary amino group of the peptide structure and the carboxyl group of lysine within a PEG derivative. This PEG derivative is a branched structure formed by conjugating 20-kDa linear PEG to lysine via a carbamate linkage. Visepegenatide is a PEGylated analog of exenatide, synthesized by substituting the C-terminal serine with a cysteine residue. The conjugation is achieved via a site-specific Michael addition between the cysteine thiol and the double bond of a maleimide-activated 23-kDa PEG moiety under optimized reaction conditions. The diverse PEGylation chemistries, molecular weights, and structural complexities of these model compounds underscore the versatility and robustness of the developed method.

This study introduces a dual optimization strategy to address the inherent response nonlinearity in the CAD of polydisperse analytes. The approach involves the systematic co-optimization of the power function value(PFV) during chromatographic separation and the power law (PL) setting in post-acquisition data processing. This dual optimization is critical for effectively linearizing the CAD response, particularly for polydisperse compounds such as PEGs.The established HPLC-CAD method is applicable to the quantification and quality control of free PEG in PEG-loxe, pegmolesatide, and visepegenatide, providing a unified analytical approach for these three PEGylated peptide pharmaceuticals. The method was comprehensively validated in accordance with applicable international and national regulatory guidelines. The evaluated parameters included specificity, precision, linearity and range, accuracy, robustness, solution stability, and the limits of detection (LOD) and quantitation (LOQ) for the corresponding drug substances and injectable formulations.

## 2 Materials and methods

### 2.1 Solvents and reagents

The following in-house retained samples were obtained for this study: a 20-kDa PEG reference standard for the determination of free PEG content in PEG-loxe (20k-PEG-lox, batch no.ZZ224P162, 97.8% purity); a 40-kDa PEG reference standard for the determination of free PEG content in PEG-loxe (40k-PEG-loxe, batch no. P1611040431509, 98.6% purity); a 20-kDa PEG reference standard for the determination of free PEG content in pegmolesatide (20k-PEG-pegmo, batch no. RS20210428, 99.6% purity); a 40-kDa PEG reference standard for the determination of free PEG content in pegmolesatide (40k-PEG-pegmo, batch no. RS20200703, 99.4% purity); a 23-kDa PEG reference standard, functionalized with a maleimide active group, for the determination of free PEG content in visepegenatide (23k-ppmalPEG-visep, batch no.Y3-009/D2401, 97.8% purity); a 23-kDa PEG reference standard, lacking functional groups, for the determination of free PEG content in visepegenatide (23k-PEG-visep, batch no.20230522, 99.4% purity). The following reagents were also obtained: polyethylene glycol loxenatide (Jiangsu Hansoh Pharmaceutical Group Co., Ltd., 2 batches); polyethylene glycol loxenatide injection (Jiangsu Hansoh Pharmaceutical Group Co., Ltd., 6 batches); pegmolesatide (Jiangsu Hansoh Pharmaceutical Group Co., Ltd., 3 batches); pegmolesatide injection (Jiangsu Hansoh Pharmaceutical Group Co., Ltd., 3 batches); visepegenatide (Chengdu Shengnuo Biopharmaceutical Co., Ltd., 3 batches); visepegenatide injection (Hangzhou Aussun Pharmaceutical Co., Ltd., 3 batches). Acetonitrile and formic acid (Thermo Fisher Scientific Co., Ltd.,Waltham, MA, USA.) were also used in this study.

### 2.2 Instruments

This experiment was conducted using a DIONEX Ultimate 3000 HPLC system (Thermo Fisher Scientific, Waltham, MA, USA).

### 2.3 High-performance liquid chromatography (HPLC)

#### 2.3.1 Test solution preparation.

Appropriate amounts of PEG-loxe, 20k-PEG-loxe, and 40k-PEG-loxe reference standards were accurately weighed, dissolved, and diluted with water to prepare solutions containing 2.0 mg/mL PEG-loxe, 40 μg/ml 20k-PEG-loxe, and 40 μg/ml 40k-PEG-loxe, respectively. These solutions were used as system suitability solutions for the determination of free PEG in PEG-loxe and its injectable formulations.

Appropriate amounts of pegmolesatide, 20k-PEG-pegmo, and 40k-PEG-pegmo reference standards were accurately weighed, dissolved, and diluted with water to prepare solutions containing 2.0 mg/mL pegmolesatide, 40 μg/mL 20k-PEG-pegmo, and 40 μg/mL 40k-PEG-pegmo, respectively. These solutions were used as system suitability solutions for the determination of free PEG in pegmolesatide and its injectable formulations.

Appropriate amounts of visepegenatide, 23k-ppmalPEG-visep, and 23k-PEG-visep reference standards were accurately weighed, dissolved, and diluted with water to prepare solutions containing 3.2 mg/mL visepegenatide, 60 μg/mL 23k-ppmalPEG-visep, and 60 μg/mL 23k-PEG-visep, respectively. These solutions were used as system suitability solutions for the determination of free PEG in visepegenatide and its injectable formulations.

PEG-loxe and pegmolesatide were accurately weighed, dissolved, and diluted with water to prepare test solutions containing approximately 4.0 mg/mL of PEG-loxe and pegmolesatide, respectively.

Appropriate amounts of visepegenatide and its stability samples (stored at 60°C for 5, 10, and 30 days; exposed to 4500 lx illumination for 5, 10, and 30 days; or conditioned at 75% RH for 5, 10, and 30 days) were accurately weighed, dissolved, and diluted with water to prepare solutions containing approximately 3.2 mg/mL of visepegenatide, serving as test solutions for visepegenatide.

Injectable formulations of PEG-loxe, pegmolesatide, visepegenatide, and stability samples of visepegenatide (exposed to 4500 lx illumination for 5, 10, and 30 days; or stored at 60°C for 5 days) were used as test solutions.

#### 2.3.2 HPLC method validation.

To evaluate specificity, blank solvent (water), an excipient blank solution (prepared according to the formulation ratio of the excipients for each PEGylated peptide), the system suitability solution, and each test solution were injected for analysis.

To evaluate precision, the 100% concentration linearity solution was injected five consecutive times, and the relative standard deviation (RSD) of the PEG peak area was calculated.

To evaluate the linearity range of the method for the determination of free PEG in PEG-loxe and its injectable formulations, appropriate amounts of the 20k-PEG-loxe and 40k-PEG-loxe reference standards were accurately weighed, dissolved, and diluted with water to prepare a series of solutions with concentrations of 0.002, 0.01, 0.02, 0.04, 0.05, 0.06, and 0.1 mg/mL. These correspond to linearity test solutions at concentration levels of 5%, 25%, 50%, 100%, 125%, 150%, and 250%, respectively.

To evaluate the linearity of the method for the determination of free PEG in pegmolesatide and its injectable formulations, appropriate amounts of 20k-PEG-pegmo and 40k-PEG-pegmo reference standards were accurately weighed, dissolved, and diluted with water to prepare a series of solutions with concentrations of 0.002, 0.01, 0.02, 0.04, 0.05, 0.06, and 0.1 mg/mL. These correspond to linearity test solutions at concentration levels of 5%, 25%, 50%, 100%, 125%, 150%, and 250%, respectively.

To evaluate the linearity of the method for the determination of free PEG in visepegenatide and its injectable formulations, appropriate amounts of 23k-ppmalPEG-visep and 23k-PEG-visep reference standards were accurately weighed, dissolved, and diluted with water to prepare a series of solutions with concentrations of 0.003, 0.016, 0.032, 0.065, 0.081, 0.097, and 0.1625 mg/mL. These correspond to linearity test solutions at concentration levels of 5%, 25%, 50%, 100%, 125%, 150%, and 250%, respectively.

To evaluate the recovery of the method for the determination of free PEG in PEG-loxe, appropriate amounts of PEG-loxe were accurately weighed, dissolved, and diluted with water to prepare an 8 mg/mL PEG-loxe stock solution. Appropriate amounts of 20k-PEG-loxe and 40k-PEG-loxe reference standards were accurately weighed, dissolved, and diluted with water to prepare mixed reference standard solutions at concentration levels of 56, 80, and 104 μg/mL. A 500-μL aliquot of each mixed reference standard solution was added to 500 μL of the PEG-loxe stock solution and mixed thoroughly to obtain spiked recovery solutions at concentration levels of 70%, 100%, and 130% of the specification limit. Each concentration level was prepared in triplicate. The solution preparation method for the recovery evaluation of free PEG in pegmolesatide was the same as that for PEG-loxe.

To evaluate the recovery of the method for the determination of free PEG in PEG-loxe injectable formulations, appropriate amounts of the 20k-PEG-loxe and 40k-PEG-loxe reference standards were accurately weighed, dissolved, and diluted with water to prepare mixed reference standard solutions at concentration levels of 280, 400, and 520 μg/mL. A 100-μL aliquot of each mixed reference standard solution was accurately measured and added to 900 μL of the PEG-loxe injectable formulation, then mixed thoroughly to obtain spiked solutions at concentration levels of 70%, 100%, and 130% of the specification limit. Each concentration level was prepared in triplicate. The solution preparation method for the recovery evaluation of free PEG in the pegmolesatide injectable formulation was the same as that for PEG-loxe.

To evaluate the recovery of the method for the determination of free PEG in visepegenatide, an appropriate amount of visepegenatide was accurately weighed, dissolved, and diluted with water to prepare a 6.4-mg/mL stock solution. Appropriate amounts of 23k-ppmalPEG-visep and 23k-PEG-visep reference standards were then accurately weighed, dissolved, and diluted with water to prepare mixed reference standard solutions at concentration levels of 91, 130, and 169 μg/mL. A 500-μL aliquot of each mixed reference standard solution was added to 500 μL of the visepegenatide stock solution and mixed thoroughly to obtain spiked solutions at concentration levels of 70%, 100%, and 130% of the specification limit. Each concentration level was prepared in triplicate.

To evaluate the recovery of the method for the determination of free PEG in visepegenatide injectable formulations, appropriate amounts of the 23k-ppmalPEG-visep and 23k-PEG-visep reference standards were accurately weighed, dissolved, and diluted with water to prepare mixed reference standard solutions at concentration levels of 538, 775, and 1000 μg/mL. A 100-μL aliquot of each mixed reference standard solution was accurately measured and added to 900 μL of the visepegenatide injectable formulation, then mixed thoroughly to obtain spiked solutions at concentration levels of 70%, 100%, and 130% of the specification limit. Each concentration level was prepared in triplicate.

To evaluate robustness, the linear correlation coefficient (*R*^2^) and the RSD of the measured concentrations of the 100% specification limit linearity solution were assessed under different chromatographic conditions, including column temperatures of 58 and 62°C and flow rates of 0.9 and 1.1 mL/min.

To evaluate stability, the 100% specification limit linearity solution was stored in the autosampler compartment at 8°C and analyzed at 0, 6, 12, 24, and 36 h. The RSD of the PEG peak area was calculated to assess sample stability.

To evaluate the limit of detection (LOD) and limit of quantification (LOQ), each PEG reference standard stock solution was diluted, and the LOD and LOQ were determined at signal-to-noise ratios of 3:1 and 10:1, respectively.

#### 2.3.3 Chromatographic conditions.

An XSelect CSH C18 column (4.6 mm × 150 mm, 2.5 μm) was used to establish the chromatographic conditions. Mobile phase A consisted of 0.1% FA in water, and mobile phase B consisted of 0.1% FA in acetonitrile. The gradient elution program for the PEGylated peptide raw material was as follows: 0–10 min, 48% → 60% B; 10–12 min, 60% → 95% B; 12–20 min, 95% B; 20–21 min, 95% → 48% B. The gradient elution program for the PEGylated peptide injection was as follows: 0–5 min, 48% B (valve switched, flow directed to waste, after 5 min valve switched, flow directed to detector); 5–15 min, 48% → 60% B; 15–17 min, 60% → 95% B; 17–25 min, 95% B; 25–26 min, 95% → 48% B. The CAD parameters were set as follows: filter, 10; temperature, 35°C. A PFV of 1.0 was applied for the quadratic calibration curve calculation. A PFV of 1.15 was separately employed for the determination of the ~ 20-kDa PEG fraction in PEG-loxe, pegmolesatide, and visepegenatide. Additionally, a PFV of 1.20 was used for the determination of the ~ 40-kDa PEG fraction in PEG-loxe, pegmolesatide, and visepegenatide. The flow rate was 1.0 mL/min, the column temperature was maintained at 60°C, and the injection volume was 20 μL.

#### 2.3.4 Data analysis.

The acquired chromatograms were processed using different PL settings based on the specific analytical purpose. A PL of 1.0 was applied for quadratic calibration curve calculation. A PL of 1.15 was used for linear regression analysis of the 20-kDa PEG content in PEG-loxe, pegmoles atide, and visepegenatide. A PL of 1.20 was employed for linear regression analysis of the ~ 40-kDa PEG content in the same PEGylated peptides.

## 3. Results and discussion

### 3.1 Optimization of PFV and PL

The power-law model is the intrinsic mathematical response model of the CAD, describing the nonlinear relationship between the injected mass and the chromatographic peak area [12]. The relationship is expressed as follows:$A=a\cdot m_{\text{inj}}^{b}$

*A*: chromatographic peak area
*m*_inj_: injected analyte mass
a: response coefficient
b: power-law exponent; b = 1 indicates an ideal linear response; b < 1 indicates a sublinear response (downward curvature); and b > 1 indicates a superlinear response (upward curvature).

In CAD-based liquid chromatography (LC) and related separation techniques, the PFV and PL are fundamental tools for optimizing quantitative analysis and addressing the inherent nonlinearity of detector responses [24, 25]. PFV is an instrument embedded parameter used for real time signal correction and is defined as the reciprocal of the power law exponent *b.*The instrument applies a power transformation to the raw detector signal in real time, ensuring that the power-law exponent of the corrected response equals unity (i.e., *b* × PFV = 1), thereby substantially broadening the linear quantification range [26].

**Fig 2:**
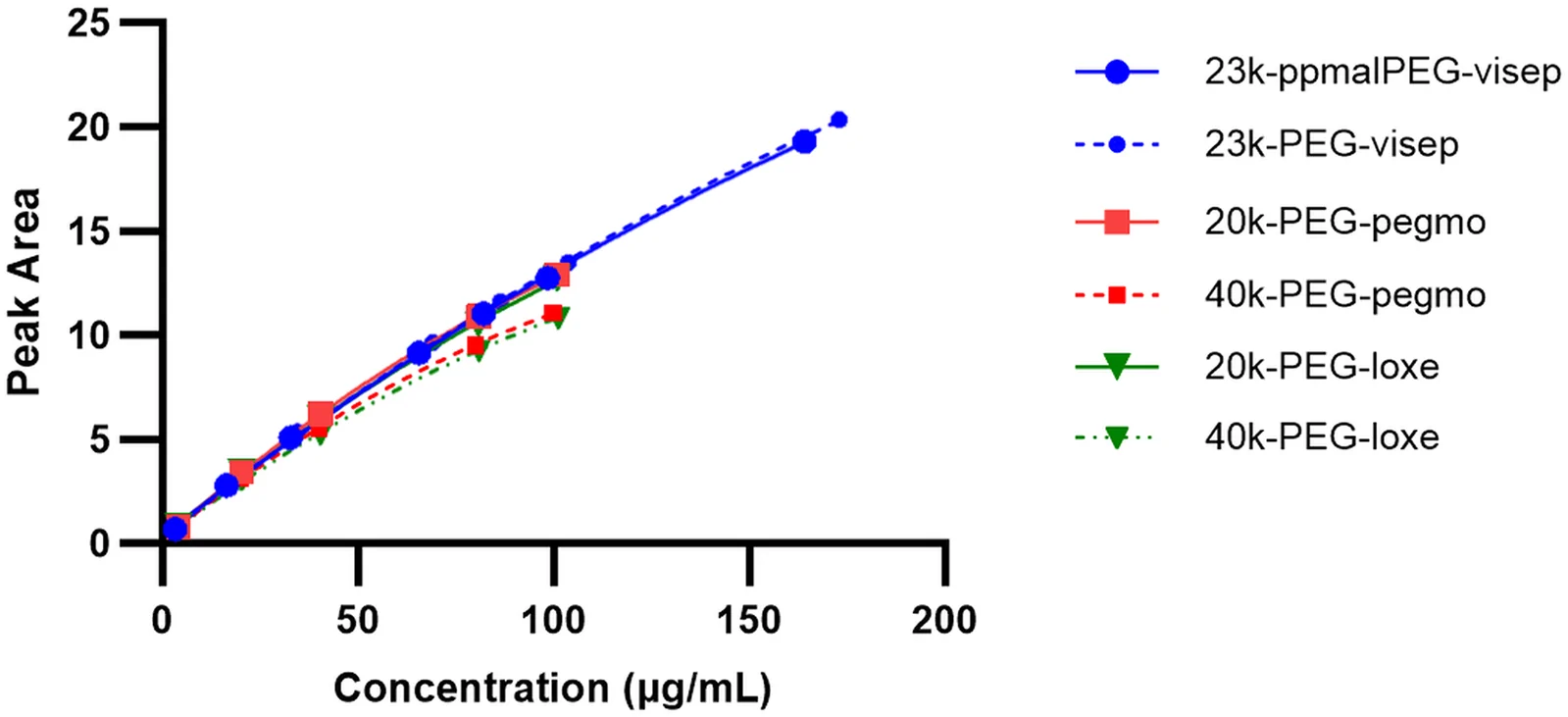
Quadratic regression curve of peak area versus PEG mass concentration at PFV = 1.0.

**Fig 3:**
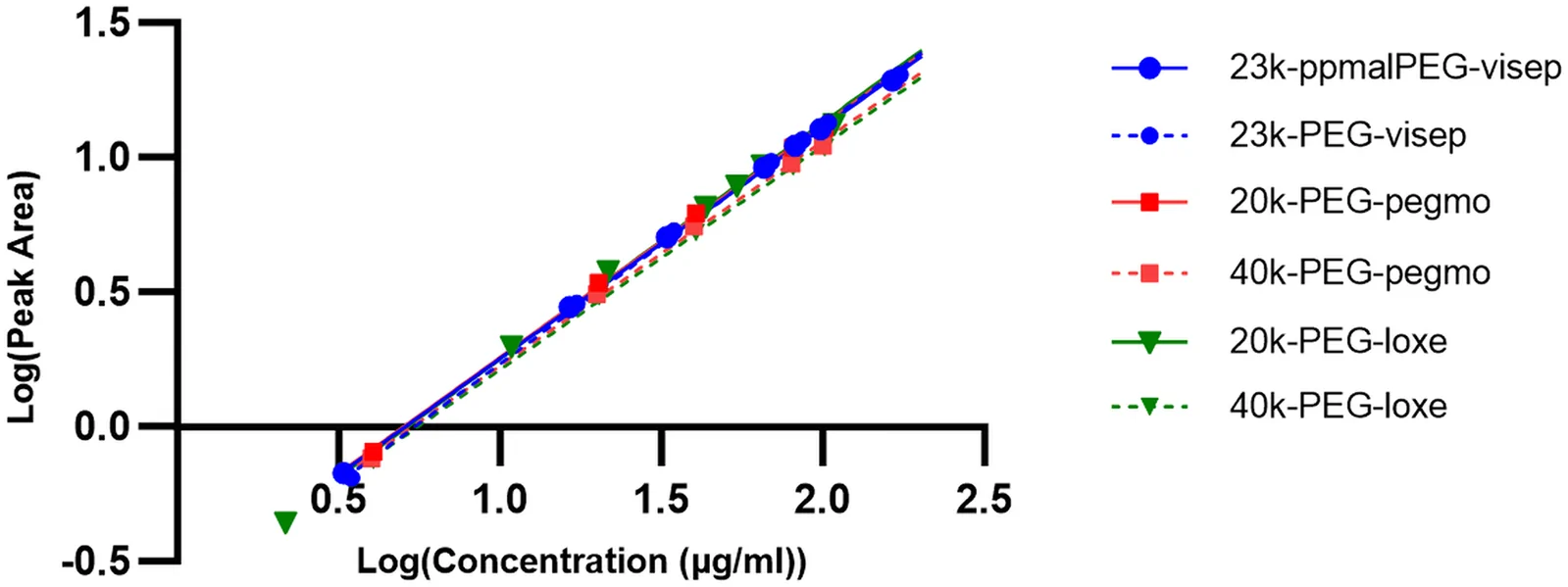
Log-log linear calibration plot of peak area versus PEG mass concentration.

This study initially acquired chromatograms of various linear PEG solutions using a PFV of 1.0 within the chromatographic system. Fig 2 presents the fitted quadratic regression curve, whereas Fig 3 presents the corresponding log-log calibration plot obtained by plotting log peak area against log concentration. The slope of the fitted line represents the detector power-law exponent *b*, and the optimal PFV was calculated as PFV = 1/*b*[12].The determined PFV values are summarized in Table 1. The mean PFV values of 1.15 and 1.20 were selected for PEGs with weight average molecular weights of approximately 20 and 40 kDa, respectively. This grouped PFV setting balances response differences among PEGs within each molecular weight category while maintaining practical applicability for routine quality control.

**Table 1:** Summary of calibration equations and PFV values for PEGs.

PEG Species	Quadratic Calibration Curve	Log-Log Equation	PFV
23k-ppmalPEG-visep	*y*=-0.0002*x*^2^+0.1454*x*+0.3494,*R*^*2*^=0.9997	*y*=0.861*x*-0.6093,*R*^2^=0.9997	1.16
23k-PEG-visep	*y*=-0.0002*x*^2^+0.1471*x*+0.2952,*R*^2^=0.9997	*y*=0.8843*x*-0.6507,*R*^2^=0.9993	1.13
20k-PEG-pegmo	*y*=-0.0004*x*^2^+0.1646*x*+0.1945,*R*^2^=0.9999	*y*=0.8626*x*-0.6054,*R*^2^=0.9993	1.16
40k-PEG-pegmo	*y*=-0.0004*x*^2^+0.1507*x*+0 2036,*R*^2^=0.9999	*y*=0.8338*x*-06072,*R*^2^=0.9990	1.20
20k-PEG-loxe	*y*=-0.0003*x*^2^+0.1522*x*+0.3016,*R*^2^=0.9993	*y*=0.8777*x*-0.626,*R*^2^=0.9980	1.14
40k-PEG-loxe	*y*=-0.0003*x*^2^+0.1392*x*+0.2525,*R*^2^=0.9997	*y*=0.8312*x*-0.6191,*R*^2^=0.9994	1.20

**Fig 4:**
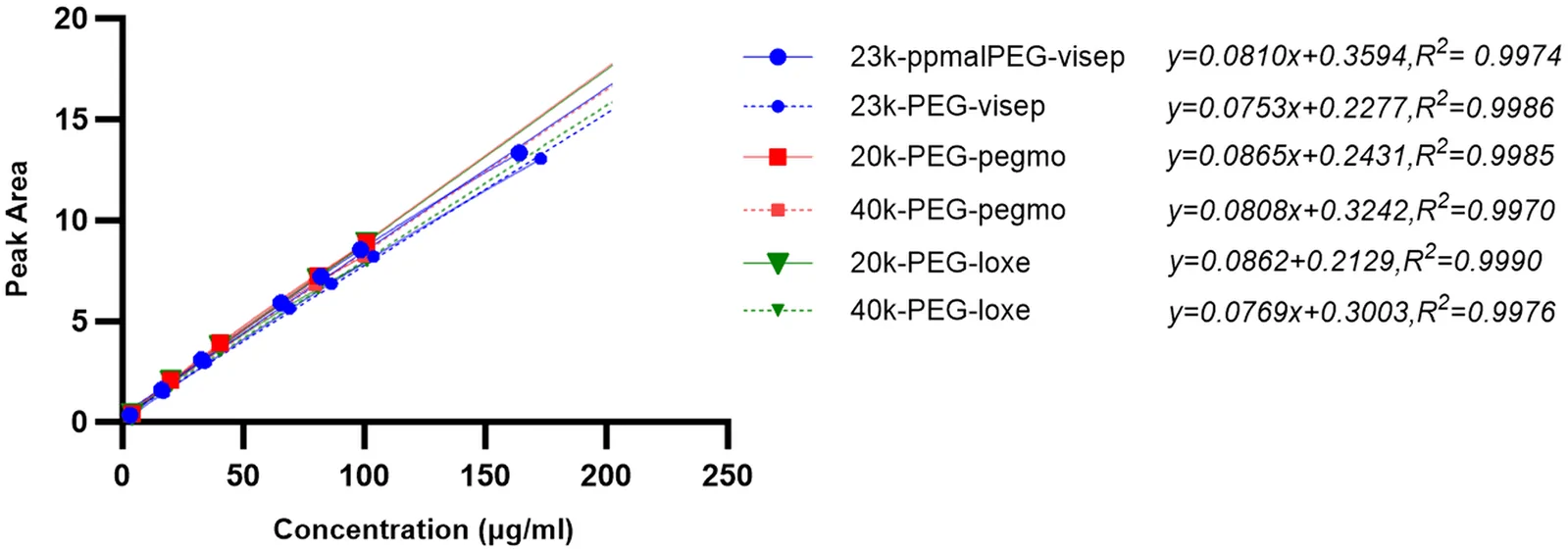
Linear relationships between PEG mass concentration and peak area after PFV correction.

During data acquisition, the PFV was set to 1.15 for the quantification of 20 kDa PEG and to 1.20 for the quantification of 40 kDa PEG. As shown in Fig 4, linear regression was performed using mass concentration as the independent variable and peak area as the dependent variable. Although all coefficients of determination exceeded 0.995, the peak area response still exhibited a slight sublinear trend with increasing mass concentration.

**Fig 5:**
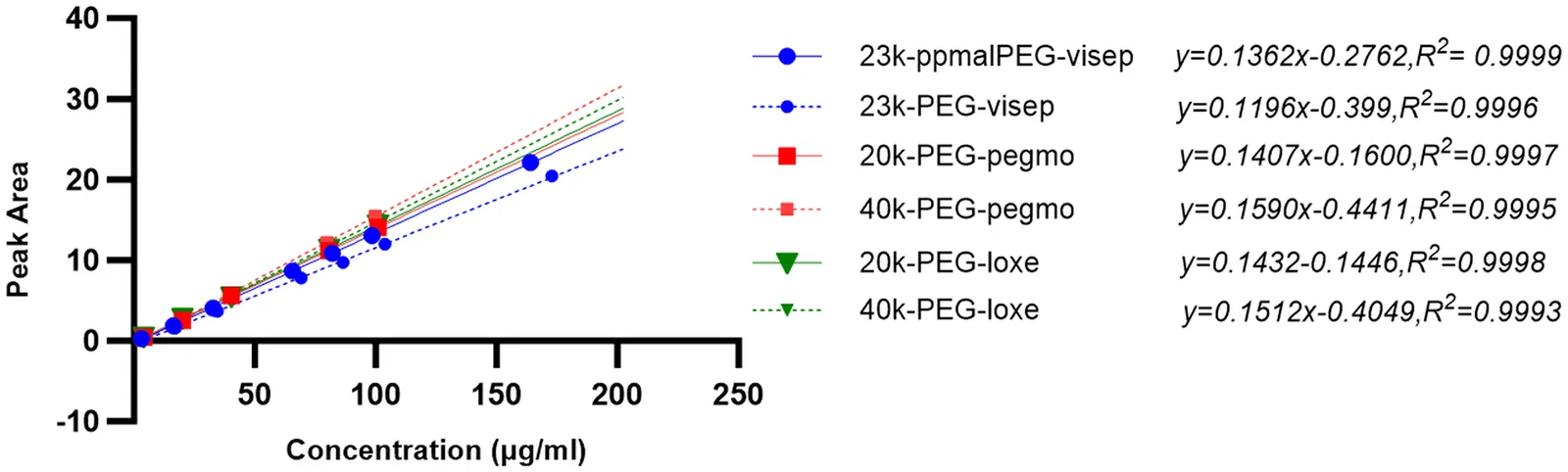
Linear calibration plots of peak area versus PEG mass concentration after PFV and PL co-optimization.

In this study, PFV was applied during data acquisition, while PL was subsequently applied during data processing. For the quantification of 20 and 40 kDa PEG, PL was set to 1.15 and 1.20, respectively. As shown in Fig 5, calibration curves were constructed by plotting peak area against mass concentration and fitting the data by linear regression. All coefficients of determination exceeded 0.999, demonstrating highly linear calibration relationships over the evaluated concentration ranges under the combined PFV and PL settings.

**Fig 6:**
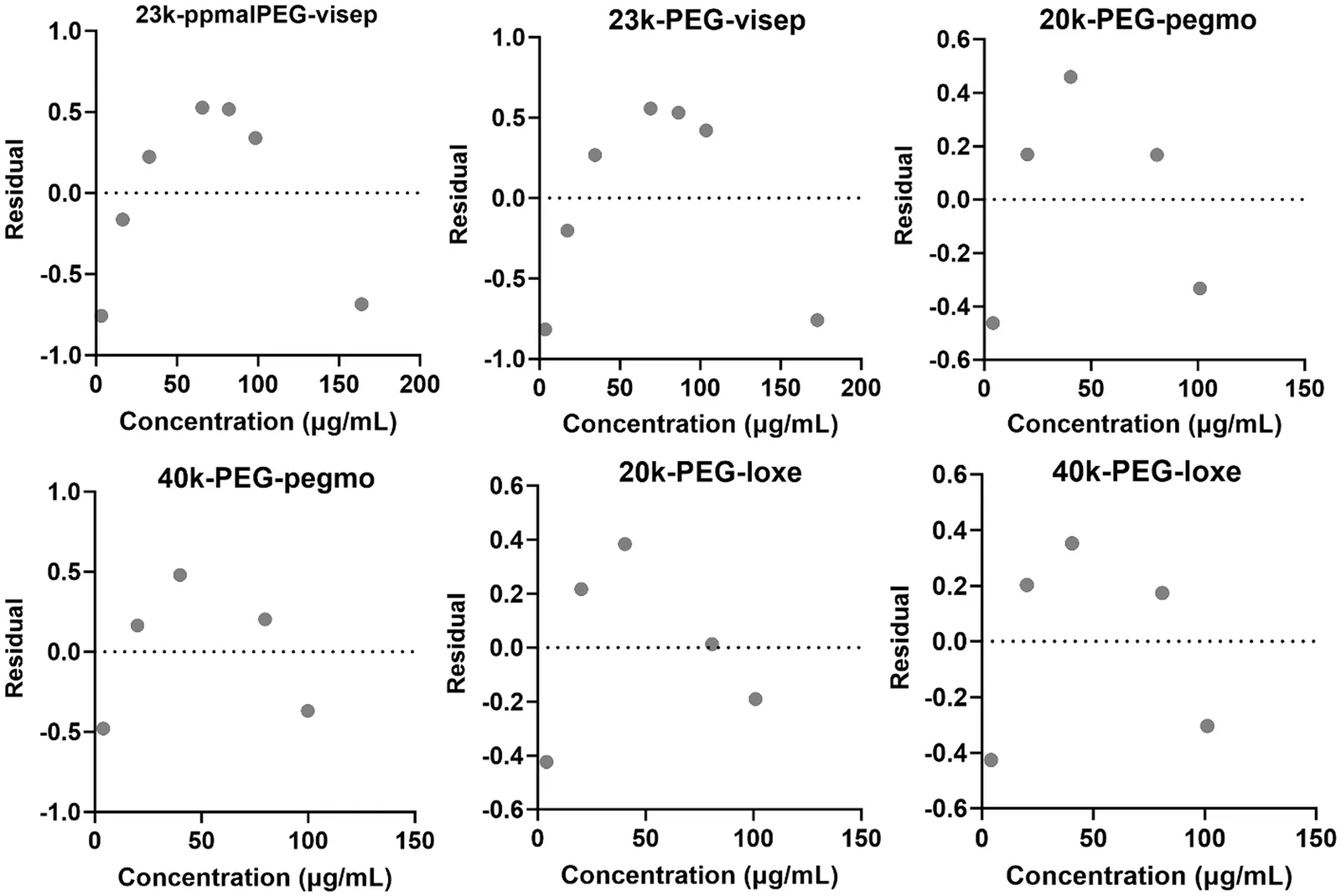
Residual plots of the linear regression for different PEGs without PFV and PL correction.

**Fig 7:**
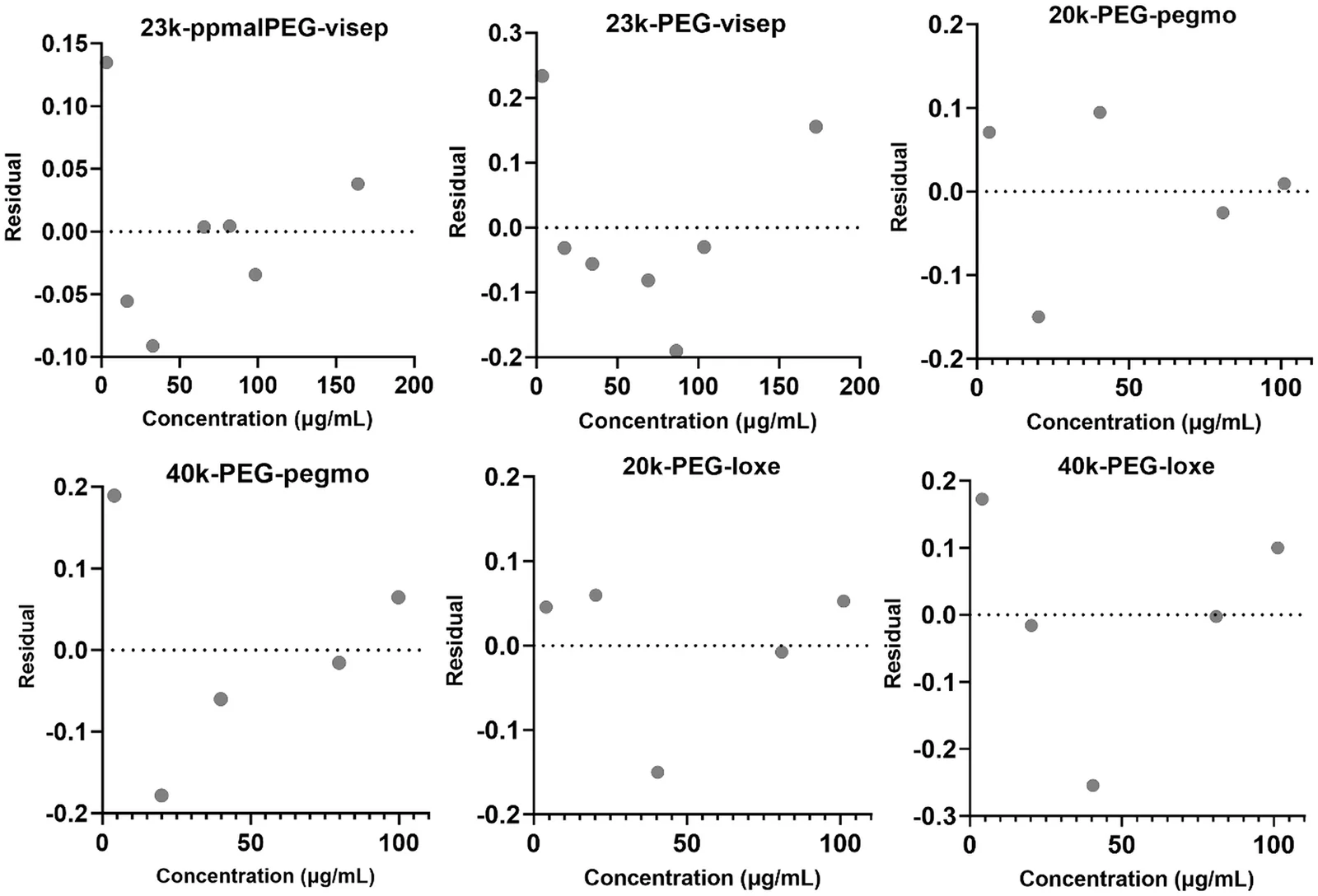
Residual plots of the linear regression for different PEGs after combined PFV and PL co-optimization.

As shown in Fig 6, under conditions where neither PFV nor PL was applied, the linear regression residuals for all PEG reference standards exhibited a characteristic inverted U-shaped distribution, with negative residuals at low and high concentrations and positive residuals at intermediate levels. This pronounced systematic trend indicates that a simple linear model cannot adequately describe the intrinsic power-law nonlinearity of the CAD under uncorrected conditions. Fig 7 presents the linear regression residual plots after combined PFV and PL correction. Following this correction, the absolute residuals were substantially reduced, the systematic inverted U-shaped trend was markedly diminished or even eliminated, and the residuals were randomly scattered around zero. These results demonstrate that this dual-parameter correction strategy effectively linearizes the CAD response, enabling reliable linear regression over the investigated concentration range.

### 3.2 Development of the chromatographic conditions

The objective of this study was to establish an HPLC–CAD method for the quantification of free PEG in three PEGylated peptide pharmaceuticals. The chromatographic separation was required not only between free PEGs of different molecular weights but also between free PEG and the corresponding active pharmaceutical ingredient (API). Because the molecular weight differences between the PEG impurities and the PEGylated APIs were relatively small, size exclusion chromatography was considered unsuitable for achieving adequate resolution. Therefore, reversed phase chromatography was selected to separate the analytes based on differences in polarity.

Gradient elution was employed by gradually increasing the proportion of mobile phase B to achieve sequential elution of the individual components. Mixtures of each API and the corresponding PEG reference standards were used as system suitability solutions to evaluate the chromatographic separation. Baseline separation was achieved for visepegenatide from 23k-ppmalPEG-visep and 23k-PEG-visep, pegmolesatide from 20k-PEG-pegmo and 40k-PEG-pegmo, and PEG-loxe from 20k-PEG-loxe and 40k-PEG-loxe. The resolution between adjacent peaks exceeded 2.0 in all cases.

The CAD is a universal mass sensitive detector for nonvolatile and partially semivolatile analytes. Its detection principle involves nebulization of the column effluent, droplet selection, solvent evaporation, charging of the resulting analyte particles by a corona discharge, and measurement of the accumulated charge using an electrometer, with the detector response being proportional to the mass of nonvolatile analytes entering the detector [27–30]. Because CAD responds to all nonvolatile components, the mobile phase should consist exclusively of volatile solvents and additives, such as formic acid, acetic acid, ammonium formate, or ammonium acetate, whereas nonvolatile salts such as phosphates and sulfates should be avoided. Otherwise, residual nonvolatile components may generate background signals, increase baseline noise, reduce sensitivity, and contaminate the detector [27–30].

For injectable formulations containing high levels of proteins, inorganic salts, buffers, or other nonvolatile excipients, a switching valve can be used to divert unwanted matrix components to waste while directing the column effluent to the CAD only during the elution window of the target analytes. Accordingly, to protect the CAD and eliminate interference from nonvolatile excipients in the injectable formulations, a separate gradient program incorporating valve switching was developed, allowing the nonvolatile excipients to be diverted to waste before the target PEGs entered the detector.

### 3.3 Methodological validation

Method validation was performed in accordance with ICH Q2(R2), *Validation of Analytical Procedures*, and General Chapter 9101, *Guidelines for Validation of Analytical Procedures*, Volume IV of the *Pharmacopoeia of the People’s Republic of China* (2025 Edition). The validation characteristics assessed included specificity, precision, linearity and range, accuracy, robustness, limit of detection (LOD), and limit of quantitation (LOQ). Sample-solution stability was additionally evaluated.

#### 3.3.1 Specificity.

**Fig 8:**
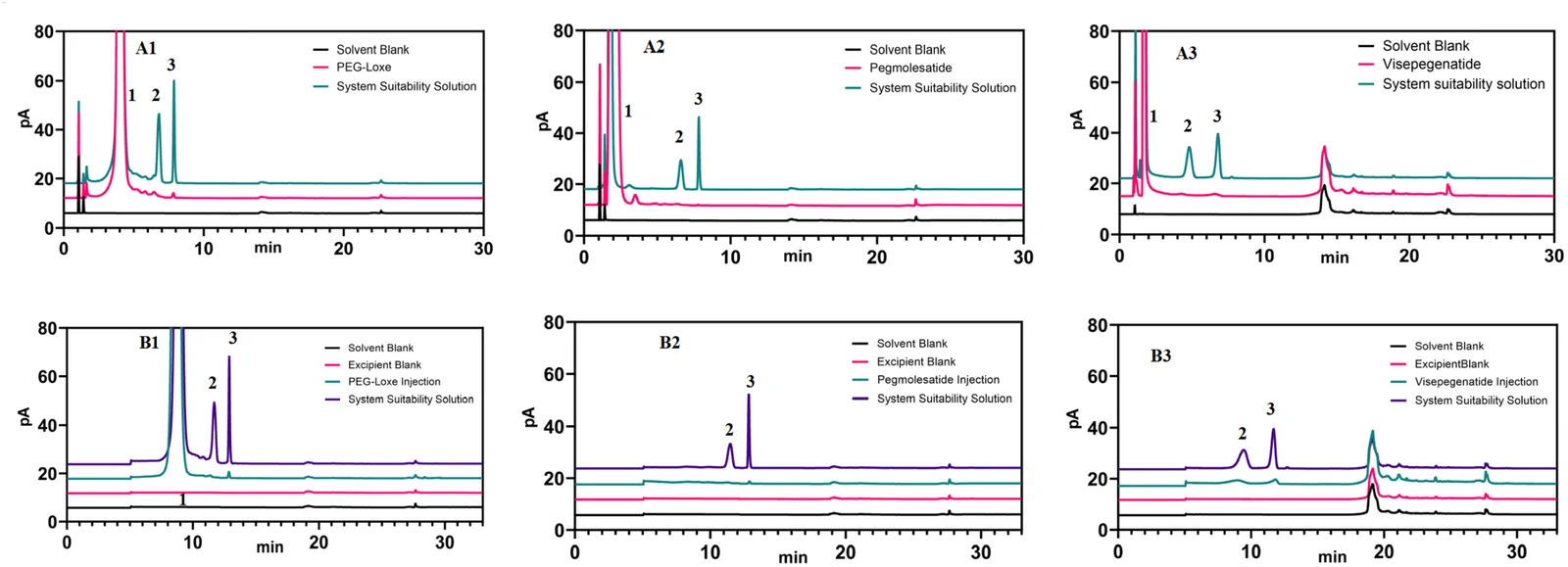
Representative chromatograms for method specificity. Panels A1–A3 show the chromatograms for the determination of free PEG content in PEG-loxe, pegmolesatide, and visepegenatide drug samples, respectively. Panels B1–B3 show the corresponding chromatograms for the determination of free PEG content in PEG-loxe, pegmolesatide, and visepegenatide injectable formulations, respectively. Peaks 1–3 in Figs. A1 and B1 correspond to PEG-loxe, 20k-PEG-loxe, and 40k-PEG-loxe; peaks 1–3 in Figs. A2 and B2 correspond to pegmolesatide, 20k-PEG-pegmo, and 40k-PEG-pegmo; and peaks 1–3 in Figs. A3 and B3 correspond to visepegenatide, 23k-PEG-visep, and 23k-ppmalPEG-visep.

The specificity of the method for determining free PEG content in PEG-loxe, pegmolesatide, visepegenatide, and their injectable formulations is demonstrated in Fig 8. Effective separation was achieved between the principal components and each PEG-related peak. No interference was observed from the blank solvent or the excipient solution, demonstrating the specificity of the method.

#### 3.3.2 Precision.

The precision of the method for determining free PEG content in PEG-loxe, pegmolesatide, visepegenatide, and their injectable formulations was evaluated. As shown in Table 2, satisfactory precision was achieved using the default PFV and PL value of 1.0, as well as the collaboratively optimized values of 1.15 or 1.20.

**Table 2:** Precision evaluation.

Method	PFV and PL settings	20k-PEG-loxe	40k-PEG-loxe	20k-PEG-pegmo	40k-PEG-pegmo	23k-ppmalPEG-visep	23k-PEG-visep
Drug substance (RSD%, *n* = 5)	1.0	1.3	1.3	0.8	1.2	0.7	0.5
	1.15/1.20	1.0	0.9	0.9	0.7	1.1	1.0
Injection (RSD%, *n* = 5)	1.0	0.9	1.3	0.8	1.5	0.8	0.8
	1.15/1.20	0.6	0.6	0.6	0.9	0.3	0.4

#### 3.3.3 Linearity range.

The linearity of the method for determining free PEG content in PEG-loxe, pegmolesatide, visepegenatide, and their injectable formulations was evaluated. Calibration curves were constructed by plotting the peak area against the concentration of each PEG reference standard. A quadratic regression equation was fitted using the default PFV and power law value of 1.0, whereas linear regression equations were fitted using the collaboratively optimized values of 1.15 or 1.20. As shown in Table 3, satisfactory linearity was achieved with both approaches.

**Table 3:** Linearity data.

Method	PFV and PL settings	20k-PEG-loxe	40k-PEG-loxe	20k-PEG-pegmo	40k-PEG-pegmo	23k-ppmalPEG-visep	23k-PEG-visep
Drug substance	1.0	*y* = −0.0004*x*^2^ + 0.1667*x* + 0.1881*R*^2^ = 0.9994	*y* = −0.0004*x*^2^ + 0.1381*x* + 0.303*R*^2^ = 0.9982	*y* = −0.0004*x*^2^ + 0.1705*x* + 0.0716*R*^2^ = 0.9996	*y* = −0.0006*x*^2^ +0.1514*x* + 0.0804*R*^2^ = 0.9995	*y* = −0.0002*x*^2^ + 0.1417x+0.3895*R*^2^ = 0.9992	*y* = −0.0002*x*^2^ +0.1471*x* + 0.2952*R*^2^ = 0.9997
	1.15/1.20	y = 0.1191*x* − 0.1504*R*^2^ = 0.9997	*y* = 0.1222*x* − 0.3195*R*^2^ = 0.9995	*y* = 0.1141*x* − 0.2604*R*^2^ = 0.9996	*y* = 0.1263*x* − 0.3932*R*^2^ = 0.9994	*y* = 0.134*x* − 0.2627*R*^2^ = 0.9998	*y* = 0.1196*x* − 0.399*R*^2^ = 0.9996
Injection	1.0	*y* = −0.0005*x*^2^ + 0.1753*x* + 0.162*R*^2^ = 0.9993	*y* = −0.0004*x*^2^ + 0.1377*x* + 0.2808*R*^2^ = 0.9984	*y* = −0.0006*x*^2^ + 0.1809*x* − 0.0173*R*^2^ = 0.9996	*y* = −0.0007*x*^2^ +0.1504*x* + 0.0983*R*^2^ = 0.9995	*y* = −0.0002*x*^2^ + 0.1422*x* + 0.3089*R*^2^ = 0.9992	*y* = −0.0002*x*^2^ + 0.157*x* + 0.181*R*^2^ = 0.9996
	1.15/1.20	*y* = 0.1204*x* − 0.1713*R*^2^ = 0.9999	*y* = 0.1235*x* − 0.3102*R*^2^ = 0.9996	*y* = 0.1207*x* − 0.2938*R*^2^ = 0.9993	*y* = 0.1326*x* − 0.3681*R*^2^ = 0.9990	*y* = 0.1282*x* − 0.4554*R*^2^ = 0.9991	*y* = 0.1164*x* − 0.3436*R*^2^ = 0.9990

#### 3.3.4 Accuracy.

As shown in Table 4, the recoveries of each PEG fell within the range of 90.0%–110.0%, with RSD values below 2.0%, under both the default PFV and PL value of 1.0 and the collaboratively optimized values of 1.15 or 1.20. These results demonstrate that both approaches enable the accurate determination of Free PEG content in PEG-Loxe, pegmolesatide, visepegenatide, and their injectable formulations.

**Table 4:** Recovery data.

Method	PFV and PL settings	20k-PEG-loxe	40k-PEG-loxe	20k-PEG-pegmo	40k-PEG-pegmo	23k-ppmalPEG-visep	23k-PEG-visep
Drug substance	1.0	100.7% (RSD = 1.4%, *n* = 9)	102.4% (RSD = 1.8%, *n* = 9)	101.0% (RSD = 1.4%, *n* = 9)	99.9% (RSD = 1.6%, *n* = 9)	103.2% (RSD = 1.0%, *n* = 9)	97.7% (RSD = 1.2%, *n* = 9)
	1.15/1.20	101.8% (RSD = 1.4%, *n* = 9)	99.8% (RSD = 1.3%, *n* = 9)	98.2% (RSD = 1.6%, *n* = 9)	98.4% (RSD = 1.0%, *n* = 9)	99.1% (RSD = 0.7%, *n* = 9)	98.2% (RSD = 1.6%, *n* = 9)
Injection	1.0	98.6% (RSD = 1.5%, *n* = 9)	101.6% (RSD = 1.6%, *n* = 9)	98.2% (RSD = 1.5%, *n* = 9)	102.0% (RSD = 1.9%, *n* = 9)	105.8% (RSD = 1.6%, *n* = 9)	100.4% (RSD = 0.8%, *n* = 9)
	1.15/1.20	101.3% (RSD = 0.9%, *n* = 9)	101.4% (RSD = 1.6%, *n* = 9)	97.9% (RSD = 1.5%, *n* = 9)	99.0% (RSD = 1.8%, *n* = 9)	99.1% (RSD = 0.7%, *n* = 9)	98.7% (RSD = 1.0%, *n* = 9)

#### 3.3.5 Robustness.

To assess robustness, the chromatographic conditions were varied, including column temperatures of 58 and 62°C and flow rates of 0.9 and 1.1 mL/min. When the PFV and PL were set to their default value of 1.0, compared with co-optimized values of 1.15 or 1.20, the linear regression equations and linear equations were fitted using mass concentration as the abscissa and peak area as the ordinate. In all cases, the *R*^2^ values were greater than 0.995. Additionally, the RSD of the measured concentrations for the 100% specification limit linearity solution was assessed under different chromatographic conditions, including the original conditions, and was found to be less than 2.0% in all cases. The results, presented in Table 5, demonstrate that the methods exhibit good robustness.

**Table 5:** Robustness data.

Method	PFV and PL settings	Chromatographic condition	20k-PEG-loxe	40k-PEG-loxe	20k-PEG-pegmo	40k-PEG-pegmo	23k-ppmalPEG-visep	23k-PEG-visep
Drug substance	1.0	Column temperature: 58°C	*R*^2^ = 0.9991	*R*^2^ = 0.9984	*R*^2^ = 0.9997	*R*^2^ = 0.9985	*R*^2^ = 0.9994	*R*^2^ = 0.9995
		Column temperature: 62°C	*R*^2^ = 0.9993	*R*^2^ = 0.9982	*R*^2^ = 0.9992	*R*^2^ = 0.9997	*R*^2^ = 0.9993	*R*^2^ = 0.9995
		Flow rate: 0.9 mL/min	*R*^2^ = 0.9997	*R*^2^ = 0.9983	*R*^2^ = 0.9997	*R*^2^ = 0.9995	*R*^2^ = 0.9995	*R*^2^ = 0.9992
		Flow rate: 1.1 mL/min	*R*^2^ = 0.9997	*R*^2^ = 0.9970	*R*^2^ = 0.9991	*R*^2^ = 0.9989	*R*^2^ = 0.9993	*R*^2^ = 0.9994
		RSD (%)	1.7	1.8	1.7	1.9	0.7	0.8
	1.15/1.20	Column temperature: 58°C	*R*^2^ = 0.9999	*R*^2^ = 0.9995	*R*^2^ = 0.9990	*R*^2^ = 0.9988	*R*^2^ = 0.9996	*R*^2^ = 0.9986
		Column temperature: 62°C	*R*^2^ = 0.9999	*R*^2^ = 0.9993	*R*^2^ = 0.9999	*R*^2^ = 0.9993	*R*^2^ = 0.9996	*R*^2^ = 0.9996
		Flow rate: 0.9 mL/min	*R*^2^ = 0.9998	*R*^2^ = 0.9996	*R*^2^ = 0.9998	*R*^2^ = 0.9996	*R*^2^ = 0.9995	*R*^2^ = 0.9992
		Flow rate: 1.1 mL/min	*R*^2^ = 0.9998	*R*^2^ = 0.9991	*R*^2^ = 0.9995	*R*^2^ = 0.9993	*R*^2^ = 0.9997	*R*^2^ = 0.9991
		RSD (%)	0.8	0.4	0.6	0.8	0.9	0.7
Injection	1.0	Column temperature: 58°C	*R*^2^ = 0.9987	*R*^2^ = 0.9970	*R*^2^ = 0.9998	*R*^2^ = 0.9982	*R*^2^ = 0.9995	*R*^2^ = 0.9997
		Column temperature: 62°C	*R*^2^ = 0.9993	*R*^2^ = 0.9986	*R*^2^ = 0.9999	*R*^2^ = 0.9982	*R*^2^ = 0.9993	*R*^2^ = 0.9996
		Flow rate: 0.9 mL/min	*R*^2^ = 0.9997	*R*^2^ = 0.9974	*R*^2^ = 0.9997	*R*^2^ = 0.9990	*R*^2^ = 0.9991	*R*^2^ = 0.9995
		Flow rate: 1.1 mL/min	*R*^2^ = 0.9993	*R*^2^ = 0.9970	*R*^2^ = 0.9996	*R*^2^ = 0.9990	*R*^2^ = 0.9995	*R*^2^ = 0.9998
		RSD (%)	1.8	1.4	1.1	1.5	1.3	0.9
	1.15/1.20	Column temperature: 58°C	*R*^2^ = 0.9999	*R*^2^ = 0.9996	*R*^2^ = 0.9995	*R*^2^ = 0.9993	*R*^2^ = 0.9993	*R*^2^ = 0.9989
		Column temperature: 62°C	*R*^2^ = 0.9997	*R*^2^ = 0.9997	*R*^2^ = 0.9996	*R*^2^ = 0.9995	*R*^2^ = 0.9990	*R*^2^ = 0.9996
		Flow rate: 0.9 mL/min	*R*^2^ = 0.9999	*R*^2^ = 0.9997	*R*^2^ = 0.9996	*R*^2^ = 0.9997	*R*^2^ = 0.9992	*R*^2^ = 0.9992
		Flow rate: 1.1 m:/min	*R*^2^ = 0.9998	*R*^2^ = 0.9994	*R*^2^ = 0.9995	*R*^2^ = 0.9995	*R*^2^ = 0.9991	*R*^2^ = 0.9990
		RSD (%)	0.5	0.9	1.7	0.9	0.6	0.6

#### 3.3.6 Stability.

The 100% specification limit linearity solution of each PEG was placed in the autosampler compartment at 8°C and analyzed at 0, 6, 12, 24, and 36 h. As shown in Table 6, when the PFV and PL were set to their default value of 1.0, compared with the co-optimized values of 1.15 or 1.20, the RSD of the PEG peak area was less than 2.0% in all cases, indicating good solution stability over 36 h.

**Table 6:** Stability data.

Method	PFV and PL settings	23k-PEG-visep	23k-ppmalPEG-visep	20k-PEG-pegmo	40k-PEG-pegmo	20k-PEG-loxe	40k-PEG-loxe
Drug substance (RSD%, *n* = 5)	1.0	1.1	1.6	1.4	1.6	1.7	1.7
	1.15/1.20	1.1	1.4	1.7	1.8	1.2	0.8
Injection (RSD%, *n* = 5)	1.0	0.6	1.1	1.4	1.1	0.5	1.4
	1.15/1.20	1.1	1.2	1.6	0.6	0.6	0.9

#### 3.3.7 Limit of detection and limit of quantification.

The LOD and LOQ were determined at signal-to-noise ratios of 3:1 and 10:1, respectively. The LOD and LOQ values underscore the high sensitivity of the method in the detection and quantification of trace levels of free PEG impurities, which is critical for meeting stringent regulatory requirements for impurity control (Table 7).

**Table 7:** Limits of detection and quantification.

Method	PFV and PL settings	Parameter (LOD/LOQ)	23k-PEG-visep	23k-ppmalPEG-visep	20k-PEG-pegmo	40k-PEG-pegmo	20k-PEG-loxe	40k-PEG-loxe
Drug substance	1.0	LOD (ng)	3.5	2.2	5.9	2.4	4.3	2.3
		LOQ (ng)	6.9	4.6	8.2	4.8	8.7	4.5
	1.15/1.20	LOD (ng)	6.9	2.2	1.4	1.6	1.4	1.5
		LOQ (ng)	13.8	4.6	2.1	2.4	2.2	2.3
Injection	1.0	LOD (ng)	6.9	2.2	13.7	4.8	2.2	2.3
		LOQ (ng)	13.8	4.6	20.6	9.7	6.2	4.5
	1.15/1.20	LOD (ng)	6.9	2.2	4.1	1.6	1.4	1.5
		LOQ (ng)	13.8	4.6	5.9	2.4	2.2	2.3

The developed RP-HPLC-CAD method demonstrated satisfactory specificity, precision, linearity, accuracy, robustness, solution stability, and sensitivity, fully meeting the acceptance criteria specified in ICH Q2(R2) and the Chinese Pharmacopoeia (2025 Edition). Collectively, these validation results confirm that the method is suitable for the reliable quantification of free PEG impurities in PEG-loxe, pegmolesatide, visepegenatide, and their injectable formulations.

In addition, to evaluate whether high drug loading would affect the quantification of free PEG, the method performance was further assessed by extending the calibration range to higher concentrations, together with direct injection and spike recovery experiments.The initial calibration ranges for the PEG reference standards corresponding to PEG-loxe, pegmolesatide, and visepegenatide covered concentrations up to 250% of the specification limit and exhibited good linearity. To further evaluate method performance at higher PEG concentrations, additional calibration standards corresponding to 300% and 400% of the specification limit were prepared for PEG-loxe and pegmolesatide. Calibration curves were established over the extended concentration range, and the concentrations of these standards were back calculated from the calibration equations to determine the back calculated recoveries. As summarized in Table 8, good linearity was maintained throughout the extended concentration range, and the back calculated recoveries at all concentration levels ranged from 90.0% to 110.0%, indicating that the method maintained satisfactory quantitative performance over the investigated high PEG concentration range, with no evidence of detector response saturation or systematic quantification bias.In addition, the test solutions were analyzed by direct injection without dilution, thereby representing the highest sample concentration and formulation matrix load encountered under routine analytical conditions. No obvious abnormalities in peak shape, retention time, or chromatographic resolution of the PEG-related peaks were observed in the chromatograms of the test solutions. Spike recovery experiments were performed directly in the injectable formulation matrix, and the recoveries at all spiking levels ranged from 90.0% to 110.0%, with RSD below 2.0%. These results indicate that the injectable formulation matrix had no appreciable effect on the quantitative determination of free PEG under the investigated conditions.

**Table 8:** Linear regression equations and back-calculated recoveries of PEG reference standards over the extended calibration range.

Specification limit level(%)	20k-PEG-loxe		40k-PEG-loxe		20k-PEG-pegmo		40k-PEG-pegmo	
	Back-calculated recovery (%)	Linear regression	Back-calculated recovery (%)	Linear regression	Back-calculated recovery (%)	Linear regression	Back-calculated recovery (%)	Linear regression
300	100.9	*y =* 0.1432*x -* 0.1446*R*^*2*^ *=* 0.9998	102.5	*y =* 0.1512*x - 0.4049R*^*2*^ *=* 0.9993	97.2	*y =* 0.1411*x -* 0.1834*R*^*2*^ *=* 0.9991	97.4	*y* = 0.1588*x* - 0.4331*R*^2^ = 0.9992
400	100.1		98.9		101.3		101.0	

Taken together, within the validated concentration range, formulation composition, and analytical conditions of this study, no appreciable adverse effects of high drug loading or formulation matrix on the quantification of free PEG were observed. The developed method is therefore suitable for the routine quality control of free PEG in high concentration PEGylated peptide injectable formulations.

### 3.4 Method application

The established and validated HPLC-CAD method was applied to determine the free PEG content in various batches of PEG-loxe, pegmolesatide, and visepegenatide drug substances and their respective injectable formulations. Furthermore, the method was used to analyze accelerated stability samples of visepegenatide, which were subjected to stress conditions including high temperatures (60°C for 5, 10, and 30 days), intense illumination (4500 lx for 5, 10, and 30 days), and high humidity (75% RH for 5, 10, and 30 days).

**Fig 9:**
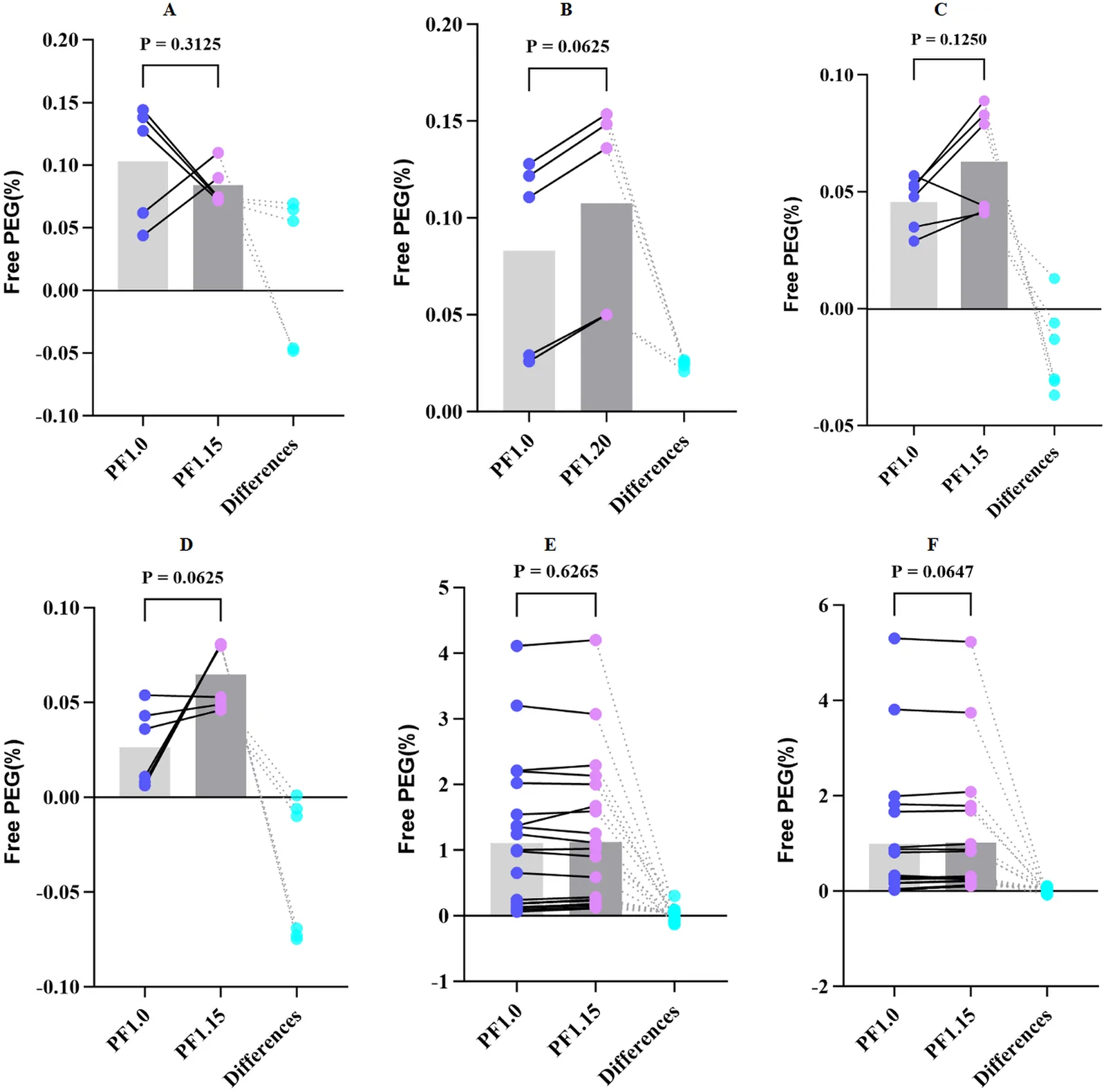
Comparison of free PEG content in PEGylated peptide samples determined by HPLC-CAD using default and co-optimized PFV and PL settings: (A) 20k-PEG-loxe, (B) 40k-PEG-loxe, (C) 20k-PEG-pegmo, (D) 40k-PEG-pegmo, (E) 23k-ppmalPEG-visep, and (F) 23k-PEG-visep.

The application of the HPLC-CAD method demonstrated its practical utility and robustness. For all tested samples, the free PEG content was successfully quantified. A Wilcoxon signed-rank test was performed to compare the results obtained using the default PFV and settings (1.0) with those obtained under the co-optimized settings (1.15 or 1.20).As shown in Fig 9, the statistical analysis consistently showed that the P-values were greater than 0.05 for all comparisons, indicating no statistically significant difference between the results obtained by the two approaches. This finding further validates the effectiveness of the co-optimization strategy in linearizing the CAD response, allowing for accurate quantification while simplifying the data processing to a linear regression model.Specifically, the analysis of accelerated stability samples of visepegenatide provided critical insights into the degradation profile of the PEGylated peptide. Under intense illumination and high temperatures, a clear and significant increase in free PEG content was observed. The ability of the method to accurately monitor these changes highlights its value in stability studies and in determining appropriate storage conditions and shelf-life for PEGylated peptide drugs. Moreover, the application of the method to different batches of drug substances and injectable formulations demonstrated good batch consistency in free PEG levels, providing a reliable tool for in-process control and final product release.

**Fig 10:**
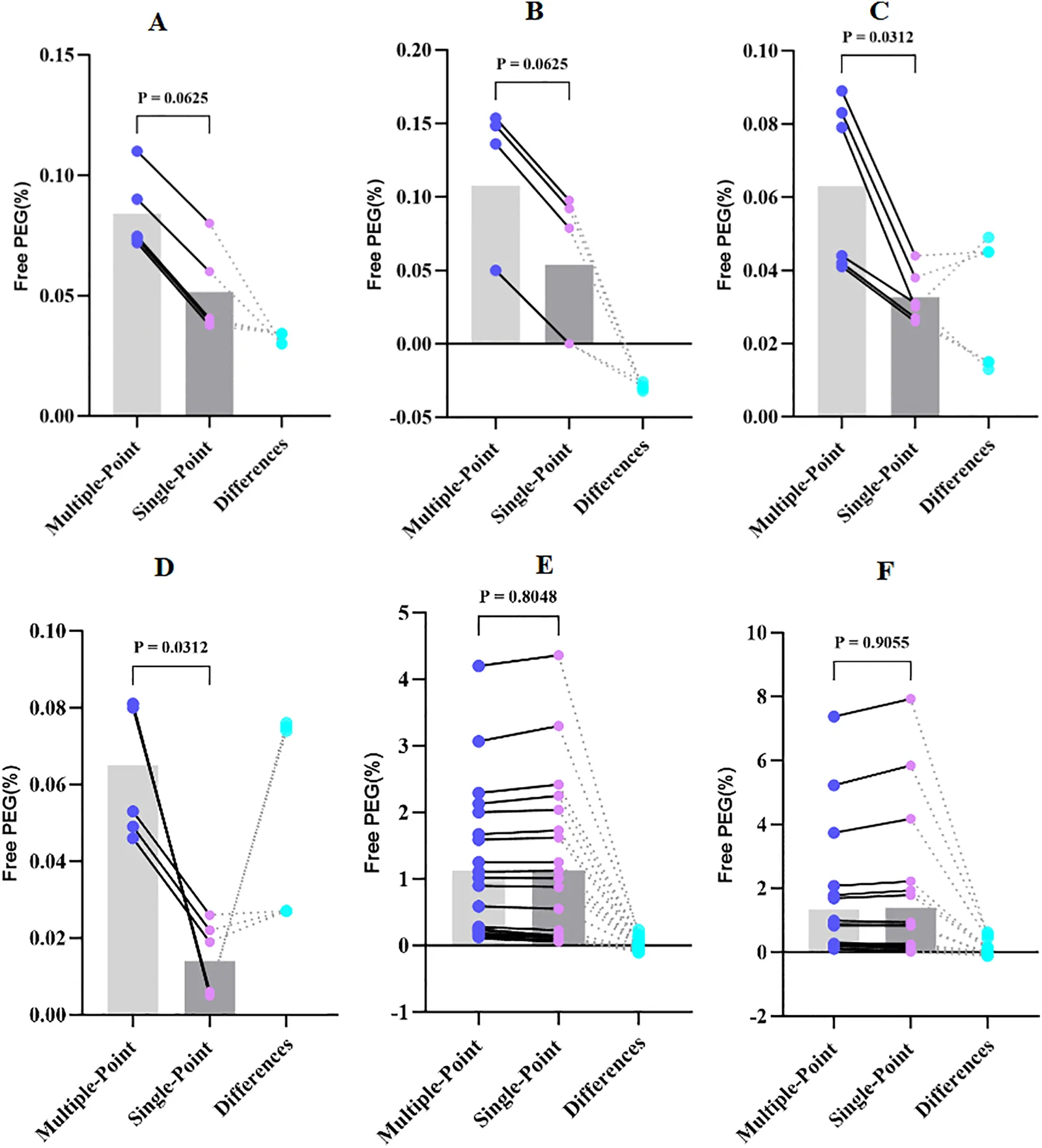
Comparison of free PEG contents in PEGylated peptide samples determined by HPLC-CAD using single-point versus multi-point external calibration with co-optimized PFV and PL settings: (A) 20k-PEG-loxe, (B) 40k-PEG-loxe, (C) 20k-PEG-pegmo, (D) 40k-PEG-pegmo, (E) 23k-ppmalPEG-visep, and (F) 23k-PEG-visep.

The free PEG contents in all samples were determined using both multi-point and single-point external calibration after the combined optimization of PFV and PL. The results from the two calibration strategies were compared using the Wilcoxon signed-rank test. As shown in Fig 10, no statistically significant differences were observed between the single-point and multi-point external calibration methods for 20 and 40 kDa free PEG in PEG-loxe, as well as the two 23 kDa species in visepegenatide (P > 0.05). These results indicate that, for these samples and within the corresponding concentration ranges, the single-point external calibration method yielded quantitative results comparable to those obtained using multi-point calibration after the combined optimization of PFV and PL.In contrast, statistically significant differences were observed between the two calibration strategies for 20 and 40 kDa free PEG in pegmolesatide (P = 0.0312). The free PEG contents in both the pegmolesatide drug substance and its injectable formulation were below 0.1%, corresponding to the lower-concentration region of the calibration curve. In this range, the contributions of the non-zero intercept, peak integration variability, and baseline noise to the overall analytical response become more prominent, resulting in discrepancies between the single-point and multi-point calibration methods for these low-level samples.

These findings suggest that the primary advantage of the combined PFV and PL optimization lies in improving the linearity of the CAD response, thereby supporting the use of a simplified single-point external calibration method within appropriate concentration ranges. Nevertheless, the applicability of single-point calibration depends on several factors, including the actual free PEG content of the sample, the intercept of the calibration curve, the concentration proximity between the reference solution and the sample, and the quantitative performance of the method in the low-concentration region. For samples containing relatively high levels of free PEG, particularly those with concentrations close to the specification limit, single-point external calibration can improve the efficiency of routine quality control. In contrast, for samples with free PEG contents near the LOQ or substantially lower than that of the reference solution, multi-point calibration or the use of a single-point reference solution at a concentration better matched to the sample is recommended to minimize quantitative bias. Overall, the improved linearity achieved through the combined PFV and PL optimization supports the feasibility of simplified single-point external calibration when the analyte concentration is close to that of the reference solution, thereby facilitating routine quality control and reducing analytical workload.

## 4 Conclusions

In this study, we successfully developed and comprehensively validated an optimized RP-HPLC method coupled with CAD for the accurate and sensitive quantification of free PEG in PEGylated peptide drugs. This method addresses the critical need for robust analytical tools in the quality control of these complex pharmaceuticals, overcoming the inherent limitations of conventional detectors such as ELSD and RI, particularly concerning sensitivity, linearity, and compatibility with the gradient elution.

A critical aspect of this method is the systematic co-optimization of the PFV (applied during the LC run) and the power law setting (applied during data processing) of the CAD. Although the CAD provides universal detection and high sensitivity for nonvolatile compounds, its inherently nonlinear response presents a significant challenge. This limitation is particularly pronounced when analyzing polydisperse analytes such as PEG, where the response complexity is further amplified [2,12 2,5 6]. Our meticulous optimization strategy, involving the determination of optimal PFV and PL, effectively linearized the detector response across a broad concentration range, as evidenced by the consistently high correlation coefficients achieved for all PEG standards. By integrating this linearization into the detection and data-processing workflow, the approach provides a methodological basis for using simplified single-point external calibration

In conclusion, the optimized RP-HPLC-CAD method developed in this study provides a sensitive, accurate, precise, and robust analytical solution for the quantitative determination of free PEG in PEGylated peptide drugs. The innovative co-optimization of PFV and PL settings effectively addresses the nonlinearity of CAD for polydisperse PEG, leading to superior analytical performance. This method provides a practical analytical tool in the quality control of PEGylated biopharmaceuticals, offering a reliable tool for ensuring product quality, safety, and regulatory compliance, while also facilitating more efficient and cost-effective routine analysis.
